# Predator-Prey Dynamics Driven by Feedback between Functionally Diverse Trophic Levels

**DOI:** 10.1371/journal.pone.0027357

**Published:** 2011-11-11

**Authors:** Katrin Tirok, Barbara Bauer, Kai Wirtz, Ursula Gaedke

**Affiliations:** 1 Ecology and Ecosystem Modelling, Institute of Biochemistry and Biology, University of Potsdam, Potsdam, Germany; 2 Ecosystem Modelling, Institute of Coastal Research, Helmholtz-Zentrum Geesthacht, Geesthacht, Germany; 3 School of Biological & Conservation Sciences, University of KwaZulu-Natal, Durban, South Africa; Dalhousie University, Canada

## Abstract

Neglecting the naturally existing functional diversity of communities and the resulting potential to respond to altered conditions may strongly reduce the realism and predictive power of ecological models. We therefore propose and study a predator-prey model that describes mutual feedback via species shifts in both predator and prey, using a dynamic trait approach. Species compositions of the two trophic levels were described by mean functional traits—prey edibility and predator food-selectivity—and functional diversities by the variances. Altered edibility triggered shifts in food-selectivity so that consumers continuously respond to the present prey composition, and vice versa. This trait-mediated feedback mechanism resulted in a complex dynamic behavior with ongoing oscillations in the mean trait values, reflecting continuous reorganization of the trophic levels. The feedback was only possible if sufficient functional diversity was present in both trophic levels. Functional diversity was internally maintained on the prey level as no niche existed in our system, which was ideal under any composition of the predator level due to the trade-offs between edibility, growth and carrying capacity. The predators were only subject to one trade-off between food-selectivity and grazing ability and in the absence of immigration, one predator type became abundant, i.e., functional diversity declined to zero. In the lack of functional diversity the system showed the same dynamics as conventional models of predator-prey interactions ignoring the potential for shifts in species composition. This way, our study identified the crucial role of trade-offs and their shape in physiological and ecological traits for preserving diversity.

## Introduction

One of the outstanding features of life on Earth is the tremendous diversity encountered at almost all hierarchical scales (e.g., at the level of functional types, species, clones and genotypes). This diversity enables ecological systems to adapt to the prevailing conditions which often buffers their responses to perturbations. Neither populations nor communities function like a mechanic watch where a change in one gearwheel is immediately, proportionally and directly transmitted to the subsequent ones. Rather, their inherent diversity enables compositional changes at lower hierarchical levels that may buffer the response at the higher hierarchical level [1]–[3]. For example, increasing grazing pressure may lead to a higher share of less edible plants which decreases grazing and the loss of plant biomass and may feed back to the biomass and community composition of the herbivores. The specific interactions are based on the functional characteristics of the interacting trophic levels, given by the functional traits of the individual species. This raises the question of how diversity and functional diversity in particular influences the mutual interplay between adjacent trophic levels or among a suite of competitors, and how this feeds back to the maintenance of diversity itself.

Developing appropriate methods for studying the effects of functional diversity poses a challenge in empirical and theoretical studies. The pivotal role of changes in the structure of trophic levels mediating the interaction with the environment and with other trophic levels is made explicit by trait-based modeling approaches. These approaches depict species (or clones, genotypes, etc.) by their functional traits and the corresponding trait values [4]–[7]. Functionally different species are represented by a continuous trait value distribution, the mean trait value indicating the strategy of the most abundant species and the variance denoting the functional diversity [8]. Altered growth conditions cause a shift in the trait value distribution reflecting an increase in the share of species better suited for the current environment. This shift can be fast when many functionally different species are present, that is, when the variability in the trait distribution is high and when the shift strongly increases the per capita net growth rate. In models, this process is indirectly traced by ‘dynamic traits’ as state variables that follow adaptive dynamics derived from underlying multi-species models [4], [9]–[12]. The approach of adaptive dynamics has been intensively used in describing evolution and co-evolution of predator and prey (e.g. [10], [13]), and adaptive behavioral dynamics (e.g. [14]). It has become increasingly popular also for studying community dynamics [5], [7], [12]. In accordance with experiments (e.g. [15]) and other modeling approaches (e.g. [16], [17]) it revealed that accounting for potential variation in trait values may strongly alter community dynamics compared to systems with fixed trait values. Hence, considering trait variation appears crucial for the understanding of the dynamics of ecological systems and their responses to perturbations. Such trait variation within an ecological entity may arise from numerous processes such as species sorting within a trophic level, shifts among clones within a population, phenotypic plasticity at the individual level and evolution. Here, we focus on trait variation predominantly arising from species shifts within a trophic level but most of our findings are also relevant for systems where other sources of trait variation dominate.

Previous models using this dynamic trait approach for describing community dynamics restricted the potential for trait variation to one trophic level (primary producers, e.g., [4], [5]). However, it is increasingly recognized that adjacent trophic levels may strongly influence each other both in respect to their diversity and dynamics (e.g. [3], [18], [19]). We therefore extend the dynamic trait approach to a predator-prey system, in which trait variation arises from a shift in species composition rather than from a shift within the genetic composition of a single species. Hence, the functional diversities of the predator and prey levels determine the speed of trait variation. Our model was inspired by interactions observed in lake plankton, where many zooplankton species graze on diverse phytoplankton which may cause changes in the species compositions of the two trophic levels, and thus the prevailing trait distributions [20]. Our system includes mutually varying functional traits, edibility of the prey (vulnerability to grazing) and food-selectivity of the predator (capture of certain prey types). We investigate the macroscopic characteristics of the trophic levels, such as their biomasses, mean trait values, and trait variances. The driving forces behind changes in the mean trait values are the trade-offs between different ecological characteristics. One reason for the existence of trade-offs in nature is physiological constraints in resource allocation, i.e., an organism that uses its resources for one function will be favored under certain conditions but cannot use the same resources again for another function [21]. Such trade-offs in the performance of physiological characteristics are widespread [5], [22]–[24]. We assume trade-offs between prey vulnerability (edibility) and maximum growth rate, prey vulnerability and carrying capacity, and food-selectivity and performance in grazing at low food concentration.

In the present study, we investigate i) how the potential of trait variation at no, one or two trophic levels influences the dynamical behavior of a two-trophic-level system and ii) the internal mechanisms that maintain functional diversity and thus the potential of trait variation. The latter includes the systematic analysis of the dependence of model results on the shape of the trade-off curves. We consider a constant environment as we are interested in internally driven dynamics rather than externally forced dynamics.

## Results

### Trait variation at no, one or two trophic levels

We analyzed different model scenarios regarding the potential of trait variation of the prey and predator trophic levels. We held the mean trait values constant (i), we allowed for trait variation within one trophic level by dynamic simulation of either the prey edibility 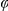 (ii) or the predator food-selectivity 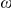 (iii), and finally we allowed for trait variation within both trophic levels, i.e., dynamic simulation of 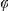 and 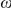 (iv).

**Figure 1:**
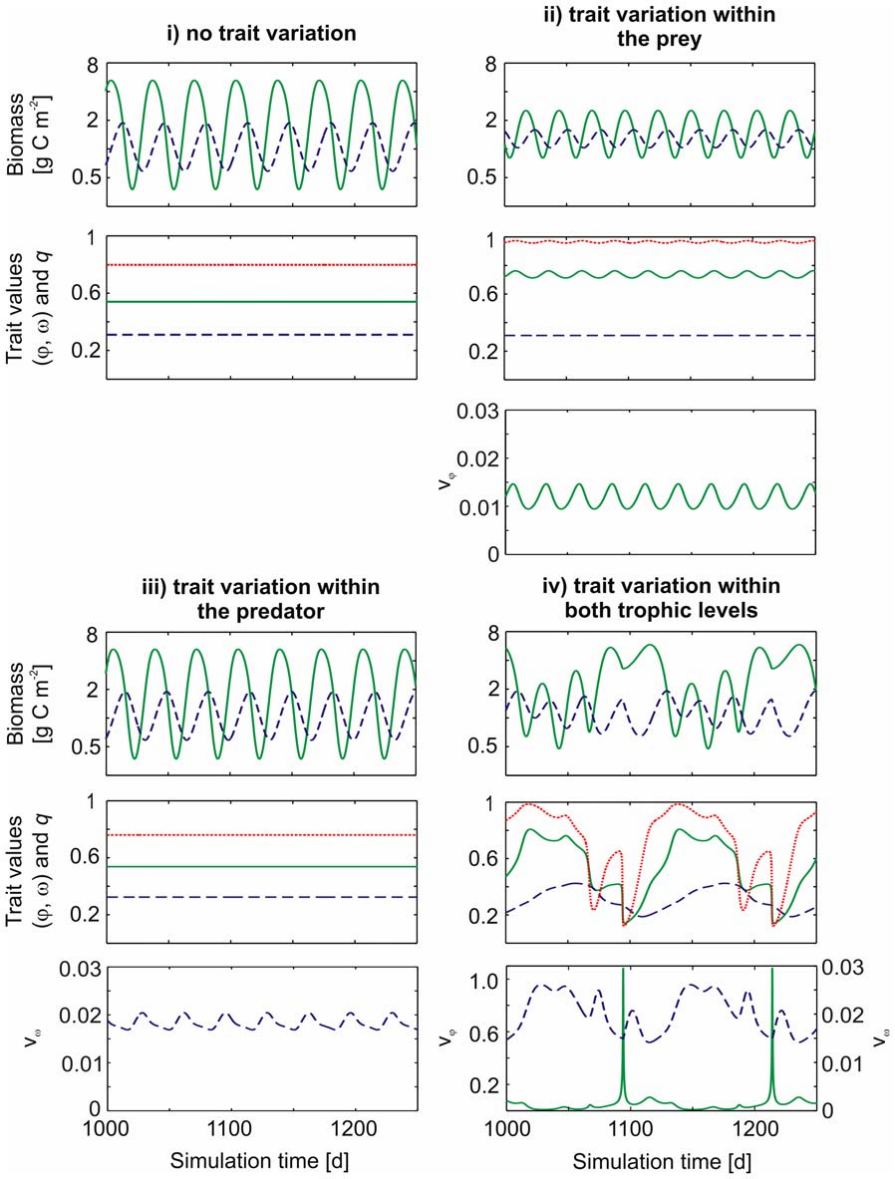
Model dynamics. Simulated prey (green solid) and predator biomass (blue dashed) (top), mean trait values, 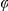 (green solid) and 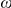 (blue dashed) and food-suitability 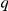 (red dotted) (middle) and variances of the trait values, 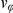 (green solid) and 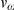 (blue dashed) (bottom, only shown when 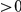), after a spin-up of 1000 days in (**i**) a model run with constant trait values for predator and prey, (**ii**) a model run with variation in 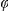, but not 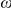, i.e., only the prey trophic level has the potential for trait variation, (**iii**) a model run with variation in 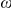, but not 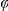, i.e., only the predator trophic level has the potential for trait variation, and (**iv**) a model run with variation in 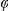 and in 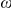, i.e., both the prey and the predator level have the potential for trait variation. Constants as in Table 1. Initial conditions for all runs were: 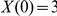, 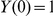, 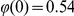, 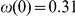, 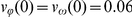. 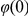 and 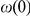 represent the mean values of d). For runs without trait variation 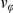 and 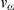 were set to zero.

- With constant trait values in both trophic levels, our model represented the classical 1-predator-1-prey situation (e.g., the Rosenzweig-McArthur model) and depicted the dynamics well established for this type of models. We obtained typical predator-prey cycles (quarter period phase lagged) when setting 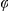 and 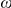 to their mean values of iii) (0.54 and 0.31) (Figure 1 A). Depending on the trait values, which control the relevant growth and grazing parameters, also fixed points appeared.
- Trait variation within the prey trophic level only, i.e., variable edibility, 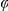, and constant food-selectivity, 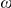, resulted in regular predator-prey cycles, but with a substantially lower temporal variability of the predator and prey biomass as compared to the dynamics without trait variation. Further, 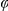 oscillated with the same frequency as the predator and prey biomass (Figure 1 B) and with a relatively small amplitude. This can be interpreted in the way that grazing by one type of predator (constant 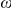 without variance) caused moderate but ongoing alternations of different prey species, and maintained functional diversity within the prey trophic level. This was observed regardless of having an external input of diversity (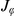) or not. Increasing predator biomass caused a decrease in prey biomass (as indicated by the typical predator-prey cycles) which, in turn, was followed by an increase in 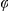. This shows that the effect of a lower prey biomass promoting fast growing prey species with lower capacity (but higher edibility) was more important than the enhanced grazing pressure favoring less edible species (cf. Figure 1 B) at the given parametrization.
- Trait variation in the predator trophic level only, i.e., variable food-selectivity, 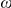, and constant edibility, 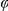, resulted in a ‘steady state’ with constant 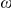. Typical predator-prey cycles with nearly the same biomass variability than without trait variation were observed (Figure 1 C). After an initial change, the mean food-selectivity (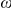) of the predator remained constant, although 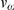 varied on a moderate level due to the invasion term. This means that small changes in diversity caused by an external input did not change the overall functional characteristics of the predator level due to lacking diversity in the prey (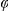 was predefined and fixed, 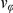 set to zero). When omitting the external input of diversity (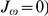, the model dynamics were the same, but 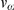 approached zero, which corresponds to a situation where one predator species out-competes the others.
- trait variation in both, the predator and the prey level, sustained ongoing cycling of the biomasses and of the mean trait values and their variances for the same parametrization as used before (Figure 1 D). The shape of the cycles differed remarkably from those without trait variation and those with trait variation restricted to one trophic level. Time periods with typical quarter-period phase lags between predator and prey biomasses (Figure 1 D, 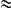
day 1010–1060, 1130–1180) alternated with periods where predator and prey were decoupled with approximately half-period phase lags and prey biomass was higher (Figure 1 D, 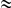
day 1070–1110, 1190–1230). Typical predator-prey cycles appeared when prey edibility was high (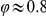), which promoted selective predators with a lower half-saturation constant keeping the prey biomass rather low. This in turn selected for a less-edible prey level. The decrease in edibility enabled the prey to escape from grazing (food-suitability strongly decreased) and to build up a high biomass. Therefore, predator and prey decoupled (Figure 1 D, day 1075, 1190). The predator level responded to the altered situation by decreasing its food-selectivity, resulting in increased food-suitability and thus increased grazing rate. This led to a further rapid decrease in prey edibility to values of 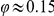. This fast shift in the mean prey trait value was supported by a high functional prey diversity at that time (Figure 1 D, day 1090, 1215). The strong decrease in edibility led to a high and less variable prey biomass for an extended period of time, despite rather high predator biomass (Figure 1 D, day 1075–1130, 1195–1250). The low edibility was linked to very low maximum growth rates of the prey (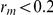 d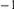). With such low maximum growth rates the prey species could not cope with the rising unselective grazing pressure when the food-selectivity of the predators further decreased. As a result, the mean edibility of the prey raised again, and this was in turn followed by an increase in food-selectivity of the predators. Increasing grazing efficiency due to a high food uptake affinity, which is linked to a high food-selectivity, finally terminated the coexistence of predator and prey at rather high biomasses and typical predator-prey cycles emerged again (Figure 1 D, day 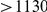).

Overall, these simulations demonstrate a strong influence of trait variation on predator-prey dynamics. Complex dynamics with feedbacks between the two trophic levels arose when both, predator and prey could respond to changing growth conditions.

### The general role of trade-offs in our model

The trade-offs (representing the relative costs and benefits of one strategy over the other) determined how the weight-specific relative net growth rates 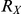 and 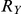, representing the fitness of prey and predator, depended on 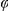, 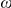, 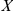 and 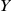. Peaks in the functions 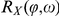 and 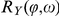 occurred when the mean trait values maximized 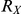 and 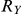, and troughs when 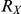 and 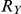 were minimized. The shape determined the direction as well as the speed of the changes in the mean trait values of the prey and predator, as both trophic levels shifted their composition in order to maximize 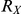 or 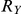, resp. The direction of the change was driven by the sign of the first derivative, and the speed of the change was affected by the second derivative as well. The latter is, by mathematical definition, negative when the function is concave (around ‘peaks’ in our set-up), and positive when it is convex (in ‘saddles’). When the trophic level was mainly composed of functional types whose traits maximized growth (i.e., the mean trait value was approaching a peak of the function 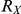) the changes in trait values slowed down because of a decline in diversity (competitive exclusion). In contrast, when the trophic level was mainly composed of functional types whose traits minimized growth (i.e., the second derivative of the 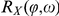 or 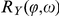 functions at the mean trait value approached a local minimum) the changes in trait values accelerated because of an increase in diversity. This correctly reflects the ecological mechanism that different functional types increase in abundance when the most abundant functional type in the trophic level suffers from large mortality or exhibits little growth.

**Figure 2:**
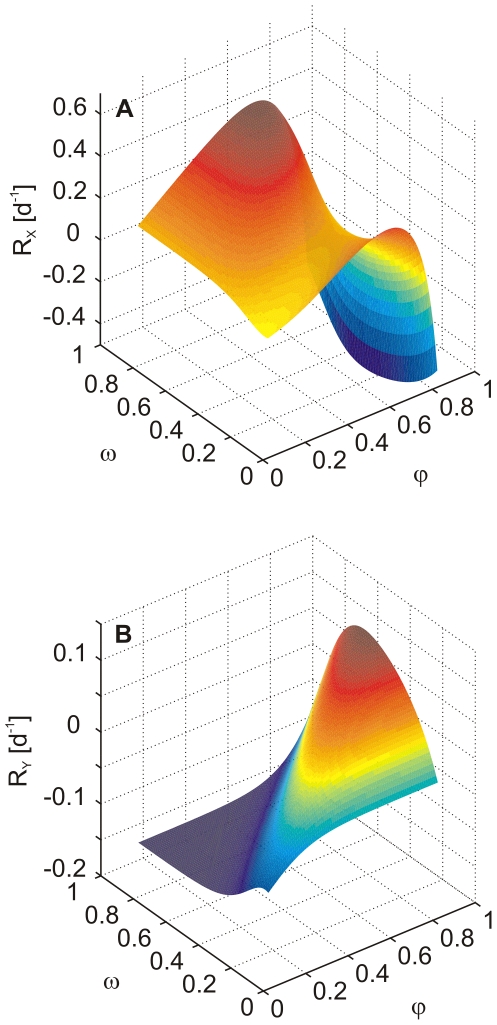
Specific net growth rates of prey and predator. (**A**) prey (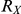, cf. Eq. (10)) and (**B**) predator (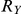, cf. Eq. (11)) in dependence of 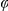 and 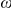. Values of the mean trait values during a standard run are 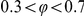 and 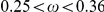. Parameters as in Table 1, prey biomass = 3 g C m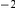, predator biomass = 1 g C m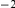.

**Figure 3:**
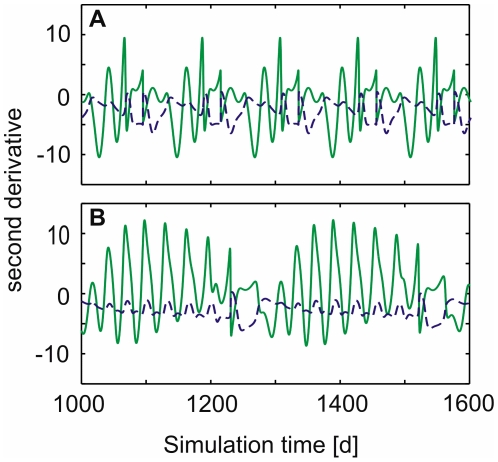
Second derivative of the specific net growth rates 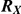
 **and** 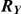 **after a spin-up of 1000 days.** 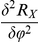 - green solid, 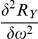 - blue dashed. (**A**) 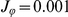 and (**B**) 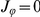.

By these means, the trade-offs determined how the diversity of the trophic level is maintained in our model. During the standard model run, values of 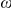 were near the optimum (Figure 2), where the function 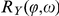 was concave. This resulted in a negative second derivative (Figure 3) implying a decrease of 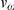 over time (cf. Eq. (6)). Hence, in the case of the predator, the Gause principle (competitive exclusion) had to be counterbalanced by an external input of functional diversity (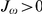) in order to maintain functional diversity. As the prey species, being subject to two opposing trade offs, alternated between highly edible and less edible forms (Figure 2), values of 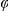 changed more strongly which meant that the second derivative determining the sign of change in 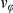 alternated between positive and negative values (Figure 3). Hence, 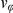 did not decline monotonically as 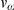 did.

### Sensitivity analysis

We ran the model with systematically altered parameter and initial values to test the robustness of our results. Using parameter values rather close to the reference values (cf. Table 1) only moderately affected the predator-prey dynamics, and with the standard parametrization no sensitivity to the initial biomass and mean trait values was found. Altering the growth and grazing parameters (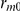, 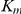, 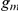, and 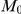) had the effects expected from classical 1-predator-1-prey models (for details see Supporting Text S2).

**Table 1:** Description and values of parameters used by the dynamic-trait model.

name	description	unit	value
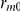	maximum prey growth rate	d 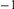	1.2
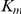	maximum carrying capacity	g C m 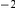	9
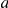	exponent for trade-off between K and 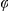 (‘cost’ parameter regarding 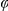 )	-	5
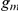	maximum grazing rate	d 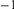	1.9
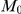	minimum half-saturation constant for grazing	g C m 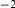	0.7
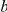	exponent for trade-off between 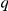 and 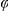 (‘cost’ parameter regarding 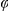 )	-	10
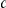	trade-off coefficient between 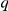 and 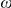 (‘cost’ parameter regarding 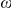 )	-	1.3
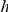	growth efficiency of predator	-	0.2
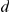	mortality rate of predator	d 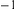	0.15
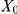	critical prey density	g C m 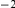	0.02
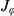	variance input of edibility	-	0.001
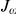	variance input of food-selectivity	-	0.001

#### Shape of trade-off functions

**Figure 4:**
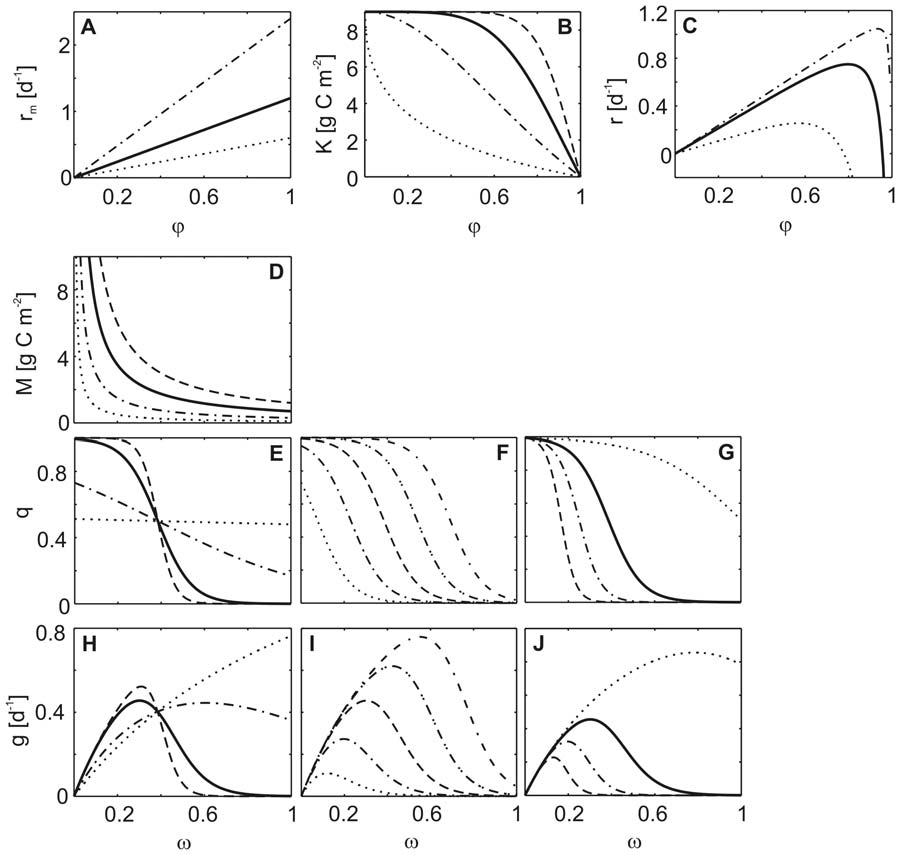
Trade-off functions between trait values of the prey (A–C) and the predator (D–J). Thick solid lines represent the functions used in the standard run. (**A**) Maximum prey growth rate 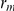, Eq. (14), for 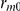 = 1.2 (solid), 0.6 (dotted), 2.4 (dot-dashed), (**B**) carrying capacity 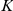, Eq. (15), for 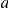 = 5 (solid), 0.5 (dotted), 2 (dot-dashed), 10 (dashed), and (**C**) gross growth rate, Eq. (7), for prey biomasses = 0.1 (solid), 1 (dotted), 5 (dot-dashed) in dependence of edibility 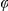. (**D**) Half-saturation constant 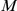, Eq. (17), for 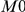 = 0.7 (solid), 0.1 (dotted), 0.3 (dot-dashed), 1.2 (dashed), (**E**) food-suitability 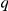, Eq. (16), for 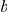 = 10 (solid), 0.1 (dotted), 2 (dot-dashed), 20 (dashed), and (**F**) 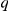 for 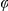 = 0.1 (dotted), 0.3 (dot-dashed), 0.5 (dashed), 0.7 (double-dot-dashed), 0.9 (dot-triple-dashed) in dependence of food-selectivity 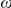, (**G**) 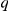 for 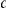 = 1.3 (solid), 0.5 (dotted), 2 (dot-dashed), 3 (dashed), (**H**) grazing rate 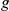, Eq. (8), for four different values of 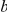 (see **E**) and 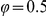, 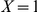 g C m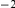, (**I**) grazing rate 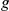 for 5 different values of 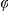 (see **F**) and 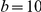, 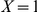 g C m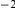 and (**J**) 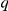 for four different values of 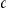 (see **G**) in dependence of 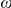. Constants as in Table 1. For details an equations see section “‘Trade-offs’” in Methods.

**Figure 5:**
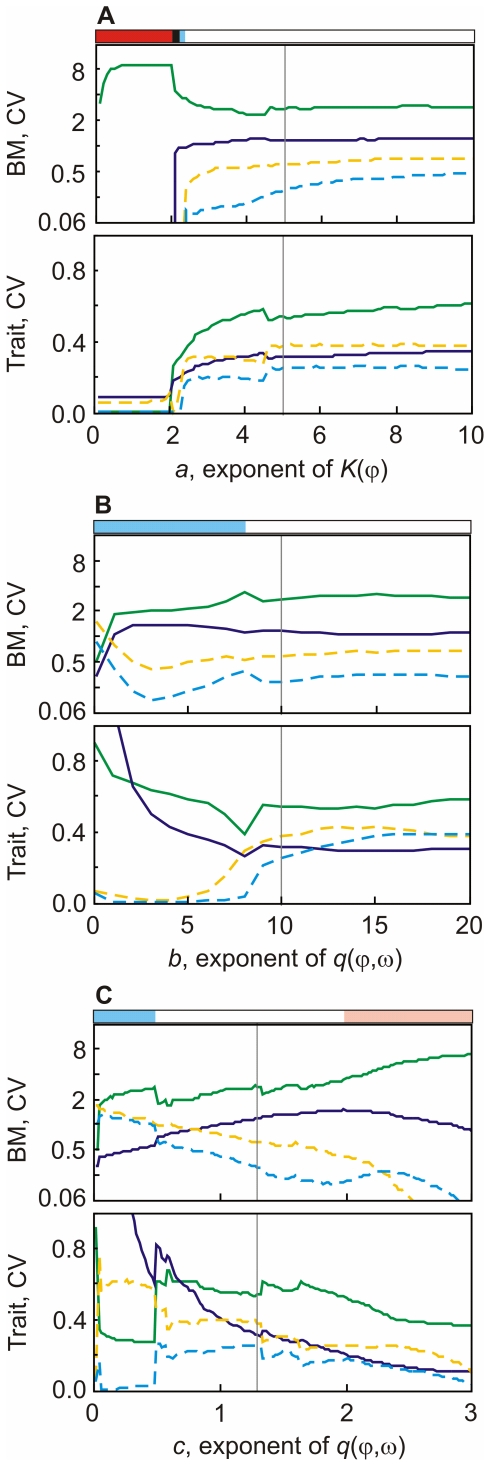
Sensitivity of the model behavior. Alterations in: (**A**) exponent 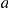 of the function 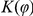, Eq. (15), (**B**) exponent 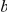 of the function 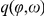, Eq. (16), and (**C**) constant 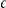 of the function 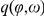, Eq. (16). Each panel comprises 2 graphs: The upper graph shows the time averaged prey (black solid line) and predator biomasses (gray solid line) (g C m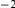, 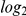 scaled) and the respective CVs (dashed lines). The lower graph shows the time averaged trait values edibility (black solid line) and food-selectivity (gray solid line) and the respective CVs (dashed lines, CVs were only calculated for biomasses 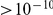). The vertical lines mark the standard parameter values as given in Table 1. The horizontal bars indicate the dynamics in the predator and prey biomass if they differ from those observed with the standard parametrization (cf. Table 1, Figure 1 D). Red bars indicate the extinction of the predator and a constant biomass of the prey at maximum carrying capacity, black bars indicate a fixed point with predator and prey coexisting (for an example see Figure 6 A). Light blue bars indicate regular predator-prey cycles, i.e., both predator and prey biomasses oscillate with the same frequency and a constant amplitude, with quarter-period phase-lags (for an example see Figure 6 B). Pink bars indicate regular predator-prey cycles with approximately half-period phase-lags (for an example see Figure 6 C). See text for details.

**Figure 6:**
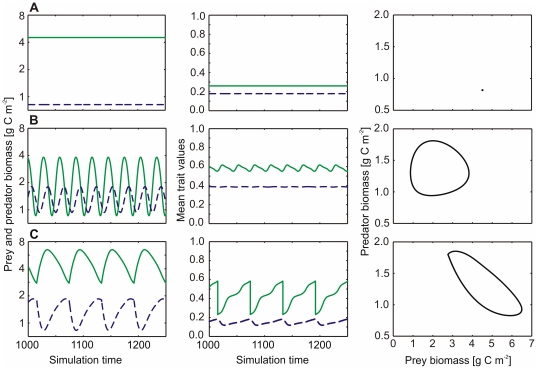
Different types of dynamic patterns obtained at different parametrization. Left: simulated prey (green solid line) and predator biomass (blue dashed line), middle: the respective trait values (green solid line for 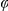 and blue dashed line for 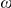), and right: phase portraits of predator and prey biomass after a spin-up of 2000 days. Model run with (**A**) 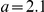, (**B**) 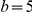, (**C**) 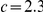. Other constants as in Table 1.

For assessing the role of the trade-offs for the entire dynamics we conducted two different types of sensitivity analysis. First, we altered the values of the trade-off parameters 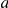, 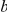 and 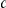 separately (see below). Second, we tested combinations of parameter changes, since the different trade-offs are interrelated (results given in Supporting Text S2). The constant 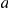 expresses the degree of non-linearity in the relation between the carrying capacity 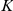 and the edibility 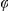 of the prey species (cf. Eq. (15), Figure 4 B). Values of 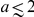 represent convex or nearly linear relationships resulting in a rather sharp decline of 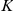 with increasing prey edibility already at low values of 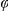. Prey species following such a trade-off strongly reduced their edibility (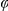 close to zero) in order to enlarge their carrying capacity 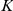 (Figure 5 A). Such less edible and, thus, slow growing prey did not sustain sufficient growth of predators to prevent extinction (Figure 5 A, note the black bar). Values of 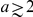 represent more concave relationships, where 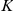 only decreases at rather high 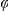 (Figure 4 B). In this case, predator and prey coexisted, either at a fixed point (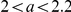, biomass and trait values constant, Figure 6 A) or at a limit cycle with ongoing alternations in species compositions (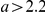, cycling biomasses and trait values). For 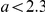 the model behavior remained similar to the standard run, that is, the limit cycle was more complex than for other values of 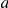.

The coefficient 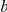 quantifies the degree of non-linearity in the relation between the food-suitability 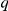 and the food-selectivity 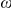 of the predator level (Eq. (16)), i.e., how much food suitability decreases with increasing selectivity. Values of 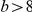 represent a rather sharp transition from maximum (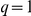) to minimum food-suitability (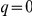) with an inflection point (Figure 5 B). Complex biomass and trait dynamics similar to the standard run were observed for these values. Low values of 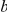 weaken the dependence of 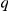 on 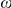 and reduce the differences between a less selective (low 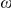) and a highly selective predator level (high 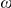) (Figure 4 E). This implies that little variability in the traits of the predator level remained, and consequently, at values of 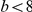 typical predator-prey cycles arose (Figure 5 B, note the gray bar, Figure 6 B).

The value of the third trade-off parameter, 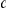, shapes the relationship between 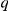 and 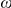 (Figure 4 G), thus, the sensitivity of a consumer's food spectrum to its selectivity, and, consequently, the typical range of 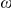 within the simulation. Very low values of 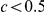 imply only a slight decrease of 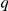 with 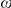, yielding unrealistically high values of 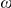 (Figure 5 C). Such high food-selectivities are linked to low half-saturation constants and resulted in typical predator-prey cycles with rather high amplitudes. For 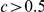, the model dynamics became more complex and similar to the standard run. High values of 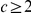 strongly favor less selective predators. In this case, predator-prey cycles were regular again (Figure 5 C, Figure 6 C).

#### The role of functional diversity

In the standard run, a small amount of variance in the trait values was added at each time step (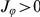, 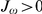) to account for ongoing invasion of species due to e.g., seed banks or dispersal. Without including this additional source of functional diversity for one or both traits, we observed a high sensitivity of model dynamics to the initial values of 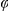 and 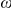. When both 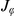 and 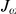 were set to zero, the trait variances 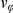 and 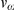 typically decreased within 500 days to very low but similar values (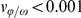 compared to 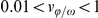 in the standard run, and the adaptive dynamics decelerated but had not faded out after 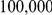 days. Periods with typical predator-prey cycles alternated with periods of constantly high prey biomass, similar to what was observed in the standard run, but at much larger time-scales because of the decelerated adaptive dynamics. That means, that the complex dynamics observed with the model indeed result from the interplay between the two trophic levels with the potential for trait variation. When adding variability to 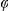 only (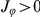, 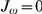), 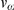 decreased to very low levels (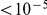 after 5000 days), whereas 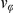 stayed 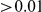. This led finally to extinction of the predator, because a rapidly adjusting prey (ongoing high diversity due to 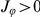) decreased its edibility (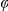) to levels which did not support a positive predator net growth. In contrast, diversity supply to the predator but not to the prey (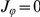, 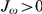) produced an alternation of quarter-period-phase-lagged cycles of different amplitudes in the biomasses, cycling in the trait values, and moderately high trait variances (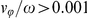), i.e., functional diversity of both trophic levels was maintained. The same holds when the variance of the predator was kept constant (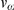 constant and 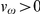) concluding that as long as the prey is grazed by a diverse predator community prey diversity is internally maintained. Mathematical this means, that 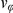 did not decline monotonically as 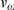 did, as the second derivative determining the sign of change in 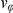 alternated between positive and negative values (Figure 3 B and cf. section “The general role of trade-offs”). This analysis shows further, that when the lower trophic level has a much higher diversity, it might exclude the higher trophic level, whereas when both have a similar level of diversity they can adjust at similar time scales, and hence coexist and show complex dynamics.

## Discussion

It is well accepted that diverse ecological systems can respond to altered growth conditions by shifts in the composition of species, clones, or genotypes and that their potential for trait variation has major consequences for their structure and dynamics [2], [13], [25]. However, most theoretical and empirical studies fall short in accommodating this key ecological feature. Our predator-prey model explicitly describes a dynamic trait for the prey (edibility 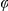), and a second one for the predator trophic level (food-selectivity 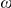), aimed to contribute to fill this gap. It simulated the mutual interplay by species shifts in both the prey and the predator, and hence revealed how changes at one trophic level may feed back to adjacent trophic levels. The model exhibited more complex dynamics than classical 1-predator-1-prey models and systems with trait variation at only one trophic level: time periods with typical quarter-period phase lags between predator and prey biomasses alternated with periods when prey and predator showed decoupled cycles apart from this typical pattern with approximately half-period phase lags. The latter was connected to fast shifts in the prey due to high functional prey diversity. During such periods of ‘decoupling’ predator and prey coexisted at rather high biomass levels as observed in laboratory experiments [26] and in the field (Lake Constance [20]). In Lake Constance, a species-rich community of small fast-growing ciliates (Protozoa) intensively grazed on several small algal species during spring [27] without inducing pronounced predator-prey cycles of the type that would be predicted by conventional predator-prey models [28]. Small sized algae and ciliates maintained high community biomasses over several weeks, i.e., numerous generations, under relatively constant abiotic conditions. In contrast, individual species biomasses strongly fluctuated and so did the relative importance of functional groups. This means that periods with a dominance of one functional type alternated with transition periods where different types were equally important [20] implying that the functional diversity is temporally highly variable. This analysis of field data indicates compensatory dynamics between functionally different species and mutual feedbacks at both trophic levels [20]. Such patterns were also successfully reflected by a multi-species model which explicitly simulated individual prey and predator species and included comparable trade-offs [17]. A detailed comparison of both approaches is in preparation (Bauer et al. in prep.).

### Relevance of trait variation for biomass dynamics and functional diversity

We represented classical 1-predator-1-prey models by the scenario where trait variation in the prey and predator was set to zero (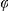 and 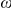 constant). As expected, this led to typical predator-prey cycles, while including trait variation remarkably changed the dynamics. Trait variation restricted to the lower trophic level(prey) significantly dampened the biomass oscillations. Such situations occur in nature when the degree of trait variation differs due to substantially different diversity or generation times at different trophic levels. For example, different levels of diversity yielded low-amplitude decoupled predator-prey cycles in experiments where prey (green algae) consisting of multiple clones responded to the altering grazing pressure of a mono-specific predator (rotifer) [26], [29]. In our model system, this case of one-sided trait variation was sufficient to sustain coexistence of different prey types and, thus, diversity, represented by ongoing alterations in the mean trait value 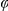, but did not yield decoupled cycles. The amplitude of changes in edibility remained relatively small, in agreement with the findings of [12] using a similar model with trait dynamics at one trophic level. The interplay of two trade-offs, one defining bottom-up forces (by a trade-off between carrying capacity and growth rate) and the other one defining top-down forces (by a trade-off between grazing vulnerability and growth rate) was essential for the maintenance of coexistence by trait variation. Only a continuous shift in the relevance of bottom-up and top-down regulation prevented that one unique, optimal strategy dominated the prey in the long-run. The reason for this ongoing shift is that the value of the prey edibility (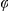), which maximizes the community fitness (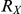), depends on both the current predator and prey biomass. On the contrary, if trait variation was restricted to the predator community, mean food-selectivity (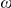, maximizing 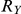) did not change over time after a short transitional phase. That is, when starting with a diverse predator community grazing on one type of prey, a single predator type always out-competed the others. This competitive exclusion is the result of the combination of the predator level following only one trade-off (bottom up, between food availability and grazing rate) and grazing on mono-specific prey.

Cycles in mean trait values implying the persistence of functional diversity in both the prey and the predator level were generated when both trophic levels had the potential for trait variation. The prey edibility, 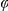, changed within a large interval depending on the prevailing predator and prey biomasses, and on the food-selectivity of the predator level. Their changes in time caused alternations in the competitive abilities of highly edible and less edible prey forms. If unselective predators exerted high grazing pressure on the whole prey level, prey biomass stayed far below the carrying capacity 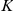, implying a low resource limitation. In this situation, the prey level was dominated by highly edible prey types with high growth rates and low 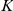 rather than less edible ones with lower maximum growth capabilities and high 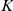. That is, high grazing pressure by unselective predators did not cause a shift in the prey level towards less edible types (low 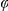) as the resulting low growth rates would cause a stronger decline in fitness than the grazing losses. This pattern is well-established for the clear-water phase in meso- to eutrophic lakes, which is characterized by a high grazing pressure (mostly by unselective filter feeders) and a dominance of fast-growing cryptomonads with high grazing vulnerability [30]. At a lower grazing pressure prey biomass increased, promoting prey species with a higher carrying capacity and less grazing vulnerability, a phenomenon also observed in nature: in meso- and eutrophic lakes phytoplankton summer blooms are formed by less edible algae with lower growth rates and high carrying capacities, which is related to their high carbon∶nutrient ratios [30], [31]. To conclude, the constraints imposed by grazing (top-down) and the carrying capacity (bottom-up) on the prey level, combined with the alternating relevance of these factors (i.e., cycling predator and prey biomasses), maintained functional diversity at the basis of the food-web.

The food-selectivity of the predator level changed rather slightly in response to changes in the prey level and in predator or prey biomasses. The strong decline of food-suitability when predators become highly selective (higher values of 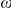) hampered a major shift in this direction (Figure 4 E, F). But still, the slight change in food-selectivity was crucial in shaping the dynamics, since without trait variation in the predators, predators and prey showed the regular, quarter-period-phase-lagged cyclescycled only in the classical way. This mechanism demonstrates the potential relevance of a delicate interplay between changes in functional traits at two trophic levels. Such small changes in the composition of trophic levels may pass unnoticed in field and experimental studies and may lead to the erroneous notion that the complex, noisy predator-prey cycles often found *in situ* are caused by external forcing rather than internal dynamics.

To conclude, changes in the structure of one trophic level promote changes of the adjacent trophic level and by this feedback to itself. These changes include alternations in trait values, i.e. certain characteristics of the trophic levels, here edibility and food-selectivity, as well as changes in functional diversity. Altogether, these feedback mechanisms drive the dynamics of the predator-prey system.

### Influence of trade-off shapes on dynamics and functional diversity

The trade-offs between different ecological characteristics are central for the temporal changes in the mean functional traits, and the shape of these trade-offs strongly influences the dynamics of real life and model systems [32]. The most relevant trade-offs and their shapes are likely to differ between different communities and systems and are empirically understudied. We addressed the question of how the trade-off shapes influence the model dynamics and which shapes were sufficient to sustain functional diversity. First, non-linearity of trade-offs (expressed by the parameters 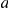, 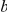 and 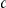) was a prerequisite to produce ongoing trait variation at both trophic levels. Second, the non-linear shapes balanced between the advantages and disadvantages of the different characteristics to sustain functional diversity and to prevent that unrealistic trait values emerged in the model. Non-linearities kept the trait values within ‘realistic’ intervals without setting fixed boundaries. This was the case for concave relationships between carrying capacity 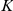 and prey edibility 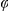 which limited the benefit to be gained via a high capacity with decreasing edibility (cf. Figure 4 B). Under a convex shape, regardless of the degree of its nonlinearity, (cf. section ‘Sensitivity analysis’), less edible prey (very low 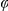 value) had a higher fitness than other types because the negative effect of increased edibility outweighed the positive effect of an increased growth rate. Therefore, less edible prey became abundant.

In the predator trophic level, a balance between advantages and disadvantages was achieved when the relationship between food-suitability 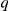 and food-selectivity 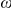 had an inflection point in combination with a positive relationship between food uptake affinity and food-selectivity (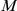-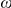 trade-off, cf. Figure 4 E, F). Selective predators (high 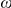) were disadvantaged by a low food-suitability in the standard run, as becoming more selective strongly reduced the probability to find adequate food (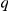 strongly decreased with 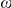). This changed with a more moderate relationship between food-suitability and food-selectivity under which selective predators became dominant due to their high food uptake affinities. Our model uses the major traits prey edibility and predator food-selectivity which is largely in line with the numerous plankton models using body size as the dominant ecological trait to quantify trophic interactions [33]–[36] because both 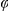 and 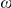 are usually correlated with body-size. Within the predator assemblage, size-independent characteristics (e.g., feeding type) determine in addition to body-size whether increasing mean prey size leads to a larger or smaller food-suitability and ingestion rate [37]. Due to size-independent traits and the high non-linearity in size-dependent functions, a mechanistic description of a size-resolving trade-off still poses a challenge. With our approach we therefore condense the many facets of size-(in)dependencies to two functionally relevant aspects, one for the prey and one for the predator.

### Maintenance of functional diversity

The predator and prey levels differed with respect to the maintenance of their functional diversity. The prey level could maintain its diversity by internal processes following two trade-offs (bottom-up and top-down) when it was grazed by different grazer types or one single type In contrast, the predator level, ruled by only one trade-off in our model, had to rely on a diverse prey trophic level and in addition, on a small external source, like migration, to keep its diversity. When feeding on monospecific prey, one type of predator excluded the others and functional diversity strongly declined when no external input of diversity was added. However, the latter did not support changes in the overall functional characteristics of the predator, i.e. food-selectivity remained constant. When feeding on diverse prey but without an external diversity input (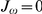), the functional diversity also declined, and the predator went extinct, when it was not able to further respond to the changing prey edibility (when 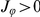). However, the decline in functional diversity occurred within several hundred days, that is, at a time scale much larger than that of the mutual feedbacks in natural plankton communities (in terms of shifts in species, morphotypes or clones) and of the seasonal cycle, i.e. the time scale of interest here. Moreover, such a decline in functional diversity fits with many closed lab experiments, where, under constant environmental conditions, diversity is often lost over time [38].

Some conventional models not using the dynamic trait approach but simulating different species with many differential equations showed maintainance of diversity without the necessity of having an external source of diversity (e.g. [17], [39]). However, in other models using the dynamic trait or adaptive dynamics approach, decreasing variance was also observed in other models using the dynamic trait or adaptive dynamic approach which was often circumvented by assuming a constant variance (e.g. [4]). However, this assumption is not realistic for natural systems which experience fast and pronounced changes in species/clones/genes etc. In our model, we added a migration term to the equation of the variance. This cannot be derived from underlying multi-species models, but reflects mechanisms important in natural systems. Furthermore, our model points to scenarios when a decline of biodiversity is expected. Functional diversity at the lower trophic level was intrinsically maintained, whereas functional diversity at the higher trophic level relied on external sources. In both cases, functional diversity is intermittently raised. These up-lifts in variance reflect concave curvatures of the respective growth functions (positive second order derivatives) which can arise due to an interplay of non-linear trait dependencies. A recent model study in evolutionary genetics [40] suggested that this “flattening” of the trait distribution can be easily produced by assuming two trade-off functions for a single trait, each controlling different growth aspects. In our model, alterations in the relevance of trade-offs follow from the dynamic nature of the predator-prey interaction.

In general, the interplay between competitive and predator-prey interactions, defined by traits and their trade-offs, determines the relative strengths and weaknesses of the different strategies (regarding edibility and food-selectivity in this study) as outlined above. Our results imply that they are also critical for the preservation of functional diversity, and for identifying communities and trophic systems where an external input is required to maintain diversity. That is, the dynamic trait approach enables predictions about the resilience of biodiversity if the main traits and trade-offs are sufficiently known, which is of remarkable importance given the serious loss of biodiversity. Functional constraints of physiological and morphological traits are yet poorly studied, but their investigation has recently gained considerable attention [6], [41], [42].

### Relation to evolutionary genetics

Models similar to ours including the potential of trait variation are often used to describe evolutionary processes where the biomass dynamics describe population dynamics, the trait values the frequency of different phenotypes and the variances the genetic variance or the probability for mutation (e.g. [43]). These studies usually keep the genetic variation constant [10], [43]. In contrast, we introduced the variance (functional diversity) as a dynamically changing variable, which is also driven by the dynamics in traits and biomasses. Also, we assumed that the distributions of the mean traits “edibility” and “food-selectivity” are uni-modal, which is a reasonable assumption for these traits when considering numerous populations (cf. section ‘Model description’), but might not be true within some populations which are e.g. under disruptive selection. The model presented here, considering the potential for trait variation at two trophic levels, also provides a framework to investigate co-evolution of predators and their prey. Typically, predator-prey co-evolution is analyzed using steady state approaches like Evolutionary Stable Strategies (ESS) or stability theory [10], [43]–[45]. Our findings suggest that cycling behavior may be more relevant in evolutionary processes as well given the conceptual and mathematical similarity between our model and predator-prey co-evolution models (e.g. [45]) and the endogenous cycling patterns arising. Previous studies on predator-prey co-evolution indicated destabilization of the food-web and high sensitivity of dynamic patterns on functional relationships among traits (reviewed by [45], [46]). This is in line with our study, where system dynamics and stability (in terms of biomass variability) was shown to depend on the shape of the trade-offs (cf. section ‘Sensitivity analysis’). The potential for trait variance may arise from numerous sources. Hence, it is an almost ubiquitous key feature of ecological systems which emphasizes the importance to account for it. The sources of trait variation likely influence details in the resulting dynamics as they determine the time scale and range of potential trait changes. Furthermore, they may influence the shape of the trait distribution which calls for more experimental and theoretical studies in this field.

### Summary

With this study we focused on investigating ecological questions such as: how functional diversity induces mutual feedback between adjacent trophic levels, and how this feeds back to the maintenance of functional diversity itself. An improved understanding of the consequences of the adaptive potential of most natural systems is necessary to more accurately predict their response to environmental change in terms of biomass dynamics and maintenance of functional diversity. We showed that resolving diversity and variation in traits (together with trade-offs) on different trophic levels may strongly shape the outcome of mathematical models. The approach presented here provides another step towards narrowing the gap between food-web models and experimental or *in situ* data.

## Materials and Methods

### Model description

The prey and predator levels are characterized by their biomasses (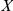, 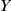 [g C m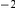]), their mean trait values (edibility 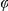, food-selectivity 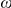 [-]), and the variances of their mean trait values (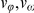 [-]), whose temporal changes are represented by six ordinary differential equations (Eq. (1)–Eq. (6)).

Traits functionally control the growth response of the plankton communities to external forcing or internal interactions. This control is described by the mathematical dependency of the net growth rate 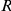 on the trait values. In our model, mean trait values represent the mean edibility of the prey level (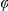) and the mean food-selectivity of the predator level (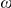). High values of 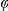 represent a prey level comprising mainly edible species, i.e., species with high vulnerability to grazing. Low values of 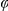 result from the dominance of less edible forms. Similarly, high values of 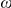 represent a predator level with mainly selective species and low values of 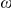 a trophic level composed of less selective species. We assume that selective predators have a specific demand for highly edible prey, whereas less selective predators can exploit most prey species although less efficiently, especially at low prey concentrations. The variances (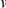) of the mean trait values represent the functional diversity present in the prey and predator trophic levels. High values of 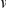 imply that a high number of functionally different species provide a large range of trait values. On the other hand, low values of 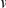 stand for the dominance of functionally similar species.

The composition of the prey and predator trophic levels, and hence, the trait distribution, may change over time by species sorting processes that reflect the consequences of competition and predator-prey interactions. This is represented by the dynamic description of the trait distribution, i.e., the mean trait values and their variances in our model. The system of the differential equations reads:

*Biomass dynamics*

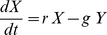
(1)

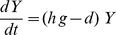
(2)with 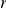 the growth rate [d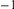], 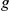 the grazing rate [d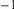] (both depending on 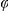 and 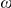, see below), 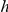 the growth efficiency [-], and 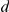 the mortality rate [d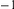].

*Trait dynamics*

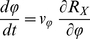
(3)

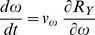
(4)

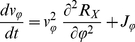
(5)

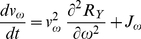
(6)with 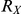 and 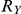 the per capita net growth rate of prey and predator (cf. Eq. (10), Eq. (11)), and 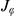 and 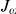 constant inputs of variance to reflect species invasions. For the derivation of the equations of the trait dynamics, see Supporting Text S1.

Parameter values are given in Table 1.

#### Biomass dynamics

The biomass dynamics (Eq. (1), Eq. (2)) are based on the equations of [47]. Growth of prey is assumed to be logistic, i.e., limited by a carrying capacity:
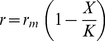
(7)with the maximum growth rate 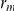 and the carrying capacity 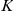. This reflects the assumption that there is a niche overlap between prey functional types within the prey trophic level and competition for common limiting resources. This assumption seems reasonable when the prey's resource base is homogeneous, as likely in pelagic systems.

Predator grazing follows a sigmoid functional response:
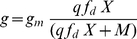
(8)

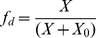
(9)where 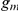 represents the maximum grazing rate, 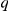 the food-suitability of the prey level as perceived by the predator level, 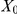 the critical prey density, and 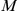 the food quantity required to achieve half-maximum grazing rates (half-saturation constant) (cf. [17], [48]). Our model simulates the dynamics of the prey and predator level as aggregates, therefore, we use the single-species form of the functional response. Food-suitability 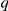 specifies the proportion of the prey species that are ingested by the predators. Our grazing function differs from the formulation 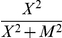 in the way that grazing is only reduced at values around 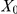, but not for prey densities considerably higher than 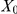. We chose a low value of 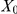 (Table 1) compared to the half-saturation constant and to average prey densities in the model. That means, the term 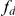 stabilizes the system at low prey densities around 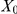, but does not have further qualitative effects on the dynamics when 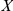 is several times larger than 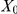. See Supporting Text S2 for model simulations with the formulation 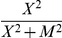.

The parameters 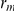, 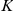, 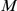, and 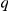 are related to the mean trait values of the prey and predator level (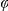, 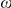, cf. section ‘Trade-offs’), and may change in time accordingly in dependence of the composition of the trophic levels. In contrast, fixed values are assigned to the parameters 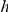, 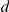, 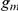, and 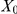 (Table 1).

#### Trait dynamics

The temporal change of the mean trait values, 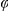 and 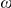, and their variances, 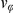 and 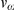 (Eq. (3)–Eq. (6)) is determined by the variances (i.e., the functional diversity) and the first and second order derivatives of the per capita net growth rates of prey and predator, 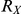 and 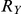, resp., evaluated at the mean trait values (Eq. (10)–Eq. (13)). 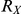 and 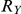 represent the fitness of the trophic levels.
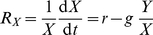
(10)

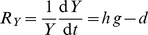
(11)

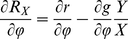
(12)

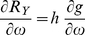
(13)

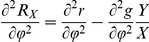


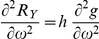



The trait dynamics (Eq. (3), Eq. (4)) is formally equivalent to the canonical equations of adaptive dynamics [10], [43]. A mathematical derivation of the equations is given in Supporting Text S1. The equations were developed in parallel in quantitative genetics and in community ecology models. They can be derived using a moment-based approximation [4], [7], [12], and here describe the more general scenario of competitive changes in a trait distribution. Trait related growth inequalities can occur in assemblages of species or even a single-clone population where individuals are able to express different ecophysiological characteristics [49]–[51].

Direction and speed of shifts in 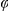 and 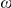 depend on how sensitive 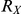 and 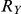 are to variations in these traits (Eq. (3), Eq. (4)). For example, under a high grazing pressure the per capita net growth rate of the prey 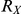 may increase by a shift towards less-edible species (lower 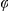) although this implies a lower maximum intrinsic growth rate (cf. section ‘Trade-offs’). This holds when the reduction in grazing losses outpaces the one in gross growth (cf. Eq. (10)). A change in the composition of a trophic level occurs fast if a change in 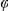 or 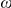 implies a strong increase in 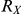 or 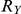 and vice versa. The values of 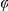 and 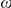 change in time in such a way that the per capita net growth rates (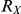 and 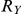) and, thus, the fitness of the trophic levels increases towards the maximum value which is possible under the prevailing growth conditions. These changes reflect a shift towards a higher share of species optimally suited for the current conditions.

The speed of shifts in the mean trait values, besides 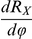 and 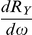, depends also on the trait variances 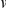 (Eq. (3), Eq. (4)), which can be understood as quantitative representation of the functional diversity [4], [44]. A high number of functionally different species provides a higher potential for trait variation than a low number of different species. Hence, only a high variance of the trait values enables fast changes of the mean trait values. Functional diversity and trait variances are not constant under natural conditions but competitive exclusion or a decline in dominant species may lead to temporal variations. In our standard model set-up, the second order derivatives of 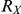 and 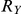, determining 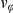 and 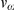, are negative near the optimal trait value (cf. section ‘General role of trade-offs in our model’). This corresponds to an increasing dominance of well adapted species and competitive exclusion of others. In this situation the potential for trait variation declines, which slows down the response of the trophic level to future alterations in the environment (Eq. (3), Eq. (4)). In natural communities, ongoing invasion of species due to e.g., seed banks or dispersal, counteract the decline in functional diversity by increasing the number of different species. 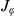 and 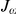 are small constant inputs of variance reflecting such species invasions in the model. We assume invasion from populations with similar trait distributions and ignore the potential but minor effect on the mean trait values (cf. Eq. (5), Eq. (6)).

The form of Eq. (5) and Eq. (6) follows from the moment-based approximation assuming that the trait distribution is normal (for details see Supporting Text S1). Also non-normal, yet uni-modal trait distributions will lead to similar equations, predicting a fast change in diversity for large variance 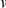 under high selective pressure (Chap. 3 in [52]). The assumption of a uni-modal trait distribution is realistic as we simulate species sorting processes where, unlike in evolutionary dynamics, disruptive selection leading to bimodal distributions does not play a major role. In plankton communities, which inspired our study, important traits like size, edibility, and selectivity are typically gradually distributed [53]–[55].

#### Trade-offs

The driving forces behind changes in the mean trait values are the trade-offs between different ecological characteristics. We introduce relations between (i) the maximum prey growth rate 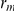 and 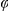, (ii) the carrying capacity 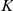 and 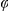, (iii) the food-suitability 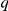 and 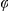 and 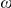, and (iv) the half-saturation constant 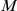 and 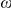.

#### Prey trade-offs

We assume a trade-off between undefended, fast-growing, nutrient demanding and less edible, slowly growing, efficient resource exploiting species which compete for common limiting resources. Thus, in our model, the maximum growth rate increases with increasing edibility of the prey level (Eq. (14), Figure 4 A) at the cost of a decreasing carrying capacity (Eq. (15), Figure 4 B).
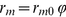
(14)

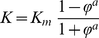
(15)

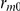
 denotes the maximum growth rate, 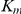 the maximum carrying capacity, and 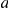, the negative relationship between 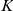 and 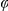 ranges from convex 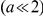 to linear 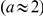 and concave 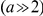 (Figure 4 B). In the standard run, we used a concave relationship (Table 1). This trade-off is based on the gleaner-opportunist dichotomy [56], [57], which is widespread in phytoplankton. Given a finite nutrient pool, the carrying capacity is inversely related to the minimum nutrient quota (N∶C, P∶C), since the lower the nutrient quota, the more biomass can be sustained. Nutrient quota are also allometrically linked to cell size [58], [59], and both determine the growth rate and quality of algae for herbivores [31].

#### Predator trade-offs

The food-selectivity of the predator level influences its feeding characteristics. Our general assumptions are that selective predators perceive only a certain part of the prey level, quantified by the food-suitability 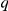 in our model, and non-selective predators perceive nearly the whole prey level, thus have a higher food quantity. This advantage is payed for by a lower food affinity, i.e. a higher half-saturation constant. Food-suitability 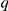 as perceived by the predator describes the match between grazing preferences and prey composition and, thus, combines characteristics of both the prey and predator level. Suitability relates to 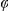 which represents various prey characteristics (e.g., morphology, N∶C, P∶C, defense structures). This motivates a non-linear relationship between 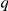 and 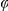 (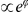). This relationship is also influenced by the suitability demand of the grazers. For a predator level consisting of highly selective grazers (high 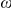), the probability that the present prey organisms match their specific demand is rather low, and, thus food-suitability decreases with 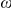 (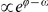). As 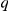 specifies the proportion of the prey level that is ingested by the predator level, it reaches values between zero and one by definition. 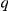 is formulated as:
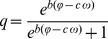
(16)with 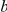 and 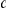 being the suitability scaling parameters (Table 1). 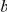 determines the steepness of the transition of 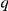 from high to low values with increasing 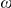 (Figure 4 E). 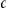 determines the range of 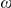 values for which 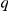 reaches high or low values, resp. (Figure 4 G). That is, 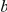 and 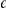 describe the “costs” for the predators of becoming selective. Food-suitability 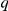 increases with decreasing values of 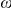 largely independently of 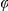 and with increasing values of 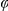 largely independently of 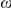 (Figure 4 E–G). This simply means that within the predator level, less selective species feed on almost all prey species equally well, and that highly suitable food is welcome to almost all grazers. On the other hand, selective predator species require highly edible prey species. Hence, a rather selective predator level (large 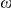) encounters highly suitable food only if 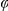 is large. When less edible prey (low 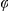) co-occur with highly selective predators (high 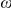) food-suitability 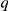 declines towards zero (Figure 4 E–G).

Food-selectivity is connected to food uptake affinities as it implies different feeding types. For example, more selective raptorial copepods with their low half-saturation constants are competitively superior in oligotrophic, algal poor systems in contrast to unselective filter feeding cladocerans such as daphnids, which are competitively superior in eutrophic, algal-rich waters (e.g. [60]). We assume, that- selective predators have higher affinities and, thus, need a lower food quantity than less selective predators to achieve half-maximum food uptake. Assuming a constant maximum ingestion rate, this is equivalent to a low half-saturation constant since food uptake affinity is given by 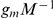 (Figure 4 D).
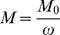
(17)

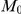
 denotes the minimum half-saturation constant at maximum food-selectivity. We used a hyperbolic function to prevent unrealistically low half-saturation constants as would result from a linear function.

The model parameters 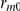, 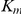, 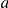, 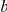, 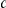 and 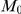 (see Table 1) were chosen such that the resulting values of 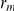, 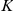, and 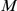 fell into the range of values for natural communities of freshwater plankton communities (e.g. [17], [61] and lit. cited therein). The relationship between the different growth and grazing parameters yields unimodal relationships between the gross growth rate (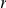) and edibility (Figure 4 C), and between the grazing rate (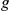) and food-selectivity (Figure 4 H–J), resp. The optimal edibility yielding the highest specific net growth rate of the prey depends on the predator and prey biomass, and on 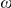. Similarly, the optimal food-selectivity where the predator level reaches its highest grazing rate, depends on the available food quantity and thus on the prey biomass and on 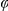 (Figure 4 I).

### Sensitivity analysis

To test the robustness of the model behavior we ran the model with systematically changed parameter values and different initial values. We altered the constant for maximum growth 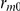, the minimum half-saturation constant 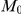, the maximum carrying capacity 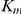, the maximum grazing rate 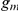, and the critical prey density 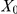 within a wide range of values (detailed results are given in Supporting Text S2). Focusing on the trade-off functions, we modified separately and simultaneously the constants (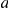, 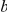, and 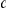) which shape the functions 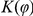 and 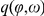 (Eq. (15), Eq. (16)), with respect to the shape and the absolute values (Figure 4 B, E).

As we did not focus on transient dynamics, we conducted the simulations for the sensitivity analysis over 5000 days, and calculated the average biomasses and trait values as well as their temporal variability, for the last 1000 simulation days. Temporal variability was assessed with the coefficient of variation (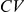, standard deviation divided by mean value). Coexistence of predator and prey with oscillating trait values, showing ongoing species shifts, is indicated by their nonzero average biomasses, traits and 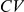
s.

Model integration was performed in MATLAB 7.x R2007b (The MathWorks, Munich, Germany).

## Supporting Information

Text S1 Moment approximation in the trait space.
(PDF)

Text S2 Further results of sensitivity analyses concerning the term for the grazing function, growth and grazing parameters, trade-offs, and initial conditions.
(PDF)
