# Supplementary material for: Predator-Prey Dynamics Driven by Feedback between Functionally Diverse Trophic Levels
Source: PLoS One. 2011 Nov 11;6(11):e27357. doi: 10.1371/journal.pone.0027357 (PMC3214039; doi:10.1371/journal.pone.0027357)
Supplement: Text S1 — Moment approximation in the trait space. (PDF) [file pone.0027357.s001.pdf]

Supporting Text S1

Moment approximation in the trait space

In our model, we resolve the trait φ for prey edibility and ω for predator selectivity. More generally, total biomass P of a functional group (either X for prey or Y for predators) collects individual contributions from group segments (organisms) sharing the same functional trait value ϕ. These segments contain biomass described by the biomass-trait distribution p_ϕ which we here assume to be a uni-variate function of ϕ only. The first order moment of the trait distribution, the trophic level trait average $\langle\phi\rangle$, follows from integrating over the product of ϕ times p_ϕ within physiologically and bio-physically attainable limits

$$\langle\phi\rangle \equiv P^{-1} \int \mathrm{d}\phi\ \phi \cdot p_\phi \qquad \text{with} \quad P = \int \mathrm{d}\phi\ p_\phi \quad . \tag{S1.1}$$

Each group segment with trait value ϕ undergoes biomass changes prescribed by the per capita net growth rate $R(\phi)$, meaning $\mathrm{d}p_\phi/\mathrm{d}t = R(\phi) \cdot p_\phi$. The distribution average of $R(\phi)$ then determines the overall biomass dynamics

$$\frac{\mathrm{d}}{\mathrm{d}t} P \;=\; \int \mathrm{d}\phi\ \cdot \frac{\mathrm{d}}{\mathrm{d}t} p_\phi \;=\; \int \mathrm{d}\phi\ R(\phi) \cdot p_\phi \;=\; \langle R\rangle \cdot P \tag{S1.2}$$

In analogy, the temporal development of the mean trait value $\langle\phi\rangle$ is determined by the trait-growth covariance.

$$\frac{\mathrm{d}}{\mathrm{d}t}\langle\phi\rangle \;=\; P^{-1}\int \mathrm{d}\phi\ \phi \cdot \frac{\mathrm{d}}{\mathrm{d}t} p_\phi \quad + \quad \left(\frac{\mathrm{d}}{\mathrm{d}t} P^{-1}\right) \cdot \int \mathrm{d}\phi\ \phi \cdot p_\phi \tag{S1.3}$$

$$= \; P^{-1}\int \mathrm{d}\phi\ \phi \cdot R(\phi) \cdot p_\phi \;-\; \langle R\rangle\, P \cdot P^{-2} \cdot \int \mathrm{d}\phi\ \phi \cdot p_\phi \tag{S1.4}$$

$$= \quad \langle\phi\, R\rangle \quad - \quad \langle\phi\rangle\ \langle R\rangle \tag{S1.5}$$

The covariance notation has occasionally been proposed also in evolutionary genetics [1]. In evolutionary genetics and (our) trophic level ecology case, it describes the (adaptive) tendency of the mean in the trait distribution to reach a state of 'uncorrelated growth', where no net gains can be achieved by moving the distribution to either lower or higher values. A positive covariance, for example, would mean that above the distribution mean trait segments grow on average faster, this way shifting the distribution function and up-lifting the mean trait $\langle\phi\rangle$, as also prescribed as an increase in time according to Eq. (S1.3). This covariance, however, is in most cases unknown and has to be estimated using available information such as the growth function $R(\phi)$ and basic distribution characteristics. It is hence re-written as a series of (central) trait moments using a Taylor expansion of R at the mean trait value $\langle\phi\rangle$,

$$R(\phi) = \sum_{n=0}^{\infty} \frac{1}{n!} \left.\frac{\partial^n R}{\partial\phi^n}\right|_{\phi=\langle\phi\rangle} \cdot (\phi - \langle\phi\rangle)^n \tag{S1.6}$$

and the simple identity $\langle a\rangle - \langle b\rangle = \langle a - b\rangle$,

$$\langle\phi\, R\rangle - \langle\phi\rangle\ \langle R\rangle \;=\; \sum_{n=0}^{\infty} \frac{1}{n!} \left.\frac{\partial^n R}{\partial\phi^n}\right|_{\langle\phi\rangle} \cdot \left(\left\langle \phi\,(\phi - \langle\phi\rangle)^n \right\rangle - \langle\phi\rangle \left\langle (\phi - \langle\phi\rangle)^n \right\rangle\right) \tag{S1.7}$$

$$= \; \sum_{n=0}^{\infty} \frac{1}{n!} \left.\frac{\partial^n R}{\partial\phi^n}\right|_{\langle\phi\rangle} \cdot \left\langle (\phi - \langle\phi\rangle)^{n+1} \right\rangle \tag{S1.8}$$

The major idea of the moment approximation is to express the higher order central moments $\left\langle (\phi - \langle\phi\rangle)^{n+1} \right\rangle$ with $n = 2, 3, \ldots$ as functions of lower order ones. For normally distributed trait values, an analytical expression of the higher moments in Eq. (S1.8) has been derived by [2] (see also "Normal distribution" in Wikipedia):

$$\left\langle (\phi - \langle\phi\rangle)^{n+1} \right\rangle = n \cdot v_\phi \left\langle (\phi - \langle\phi\rangle)^{n-1} \right\rangle \tag{S1.9}$$

Independently from the underlying distribution function, the moment closure Eq. (S1.9) is exact for $n = 0, 1$. In the case $n = 1$ it provides the definition of the variance v_ϕ as second order central moment of the trait distribution.

$$v_\phi = \left\langle (\phi - \langle\phi\rangle)^2 \right\rangle = \left\langle \phi^2 \right\rangle - \langle\phi\rangle^2 \quad . \tag{S1.10}$$

For non-Gaussian distributions and higher values of n we refer to the Schwarz inequality leading to a lower estimate for the moment closure

$$\left\langle (\phi - \langle\phi\rangle)^{n+1} \right\rangle \geq v_\phi \left\langle (\phi - \langle\phi\rangle)^{n-1} \right\rangle \tag{S1.11}$$

When the order (n) grows, the left hand side of the inequality in general gets increasingly larger than the right hand side. An exceeding inequality is partially captured by the proportionality factor n (equal to the intermediate exponent of the closure) in Eq. (S1.9). This equation is therefore taken as a first approximation for the moment closure also in the more general case of non-Gaussian trait distributions. Its insertion into Eq. (S1.8) and, for clarity, the usage of a new order index $n' = n - 1$, leads to the central equation of the mean trait dynamics also used in our model:

$$\frac{\mathrm{d}}{\mathrm{d}t} \langle\phi\rangle = v_\phi \sum_{n=1}^{\infty} \frac{n}{n!} \left.\frac{\partial^n R}{\partial \phi^n}\right|_{\langle\phi\rangle} \cdot \left\langle (\phi - \langle\phi\rangle)^{n-1} \right\rangle \tag{S1.12}$$

$$= v_\phi \sum_{n'=0}^{\infty} \frac{1}{n'!} \left.\frac{\partial^{n'} (\partial R / \partial \phi)}{\partial \phi^{n'}}\right|_{\langle\phi\rangle} \cdot \left\langle (\phi - \langle\phi\rangle)^{n'} \right\rangle \tag{S1.13}$$

$$\overset{Eq.\,(S1.6)}{=} v_\phi \left\langle \frac{\partial R}{\partial \phi}(\phi) \right\rangle \tag{S1.14}$$

Temporal changes of $\langle\phi\rangle$ follow an averaged gradient of the relative growth rate. The speed of such shifts is also proportional to the variance of the trait distribution. Functional diversity thus enhances the ability of a trophic level to adaptively respond to changes in the environment, the latter described by alterations in R.

The approximation made in the moment closure only infers a small error for non-Gaussian distributions. Figure S1.1 shows an example for the right-skewed Gamma distribution function and a functional dependency $R(\phi)$ which is nearly identical to the uni-modal function of consumer growth on selectivity used in this study. While a normal distribution is fully captured by the closure approximation, the greater value of higher order terms (e.g., skewness as the third order moment) of the Gamma distribution together with the negative value of the 2nd order derivative (close to the growth optimum with $\partial R/\partial \phi = 0$) lead to an overestimation of Eq. (S1.12) compared to the original series Eq. (S1.8). The cumulative deviation, however, converges to a relatively small value owing to the negligible value of higher order derivative terms in the expansion of $R(\phi)$. A final deviation in the range of 1 to 10 %, seems to be insignificant in relation to the often much higher uncertainties in growth functions and parameters. The recent study of Maerz et al. [3] has demonstrated that the 2nd order moment approximation works very well even in the case of skewed distributions and highly non-linear interaction functions.

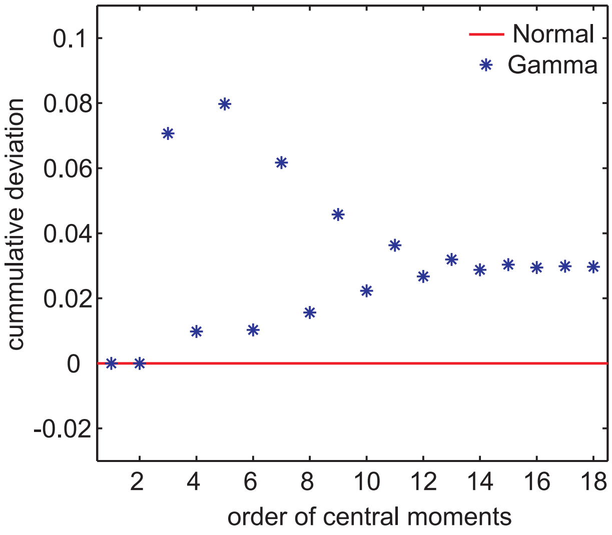

Figure S1.1. Cumulative deviation between the exact formulation Eq. (S1.8) of the trait-growth covariance and the approximation Eq. (S1.12) plotted over the order of the central moments (series elements). The lack of error for normal distributions (red line) is contrasted to the case of a Gamma distribution ($k = 3$), with $p_\phi = c^{-1}\phi^2\,\mathrm{e}^{-\phi/\phi'}$ ($c = \Gamma(3)\cdot\phi'^3$, $\phi' = \langle\phi\rangle/3$). For the relative growth rate depending on the trait value ϕ, a non-linear function with analytically known higher order derivatives is chosen: $R(\phi) = \phi\mathrm{e}^{-\phi}$. This function closely resembles the consumer growth rate Eq. (8) depending on selectivity ω through Eq. (16) and Eq. (17). The absolute value of the growth function derivative $\partial R/\partial\phi$ varies from 1 (at $\phi = 0$) to -0.14 (at $\phi = 2$).

This result and the overall derivation of Eq. (S1.14) show that the numerical error in the trait dynamics will only be considerable if both, higher order derivatives of R and higher order moments (e.g. related to skewness and kurtosis of the trait distribution) will attain large absolute values at the same time. Mathematically, the condition would appear as non-smooth curvatures in the trait space of both, the growth and the distribution function. This condition is not approached by our study since we assume a skewed, uni-modal trait distribution similar to the Gamma distribution, and non-linearities in net growth rates of prey and consumers are not exceeding the one in the test case. In applications of the moment approximations, however, where the condition is met the trait variable may have been ill defined. In ecological terms, this situation would represent a trophic level (in the one-dimensional trait space) with (1) strong specialization in a well defined niche, i.e., high abundance within a specific range of trait values and (2) extreme diversity along one broad or many niches, i.e. lower, but non-negligible abundance in other ranges of trait values, both realized at the same time. If this scenario occurs at all, it can be expected to be only of transitory type. As a solution, a non-linear transformation of ϕ to a (normally distributed) trait ϕ' transfers the problem to re-calculating growth-trait derivatives.

Even for Gaussian–like distributions, high non-linearity in the growth function resulting in substantial higher order derivatives can deter precise determination of ensemble means such as $\langle R\rangle$ in Eq. (S1.2) or $\langle\partial R/\partial\phi\rangle$ in Eq. (S1.14). Growth performances of organisms with trait values much distant from $\langle\phi\rangle$ do usually not equal the group average $\langle R\rangle$. Due to these deviations, the ensemble mean of interactions functions like $\langle R\rangle$ differ from the function evaluated at $\langle\phi\rangle$. To handle such cases, a simplified version of the Taylor expansion shown above in Eq. (S1.8) provides a second order correction. For the ensemble mean of net growth rate in Eq. (S1.2), the correction is proportional to the trait variance [2]:

$$\langle R\rangle \simeq R(\langle\phi\rangle) + \frac{1}{2}\,v_\phi\cdot\left.\frac{\partial^2 R}{\partial\phi^2}\right|_{\langle\phi\rangle} \tag{S1.15}$$

The variance v_ϕ, alike the mean trait, is evolving in time when differential growth conditions reshape the trait distribution. A mathematical formulation of this variance dynamics follows from repeating the calculus from Eq. (S1.3) to Eq. (S1.12). Moment exponents are, however, elevated by one order, what can be dealt with by a doubled application of Eq. (S1.9).

$$\frac{\mathrm{d}}{\mathrm{d}t} v_\phi = \left\langle \phi^2 R \right\rangle - \left\langle \phi^2 \right\rangle \langle R \rangle - 2 \langle \phi \rangle \frac{\mathrm{d}}{\mathrm{d}t} \langle \phi \rangle \tag{S1.16}$$

$$\simeq v_\phi^2 \left\langle \frac{\partial^2 R}{\partial \phi^2} \right\rangle \tag{S1.17}$$

An alternative and much simpler way to derive Eq. (S1.17) starts from the approximation of the average growth rate in Eq. (S1.15) where one can treat the variance v_ϕ as independent trait variable. As this variable affects the (average) net growth rate $\langle R \rangle$, its dynamics can be given according to the central trait dynamics Eq. (S1.14). Derivation of the right hand side of Eq. (S1.15) with respect to v_ϕ and calculation of the "variance in the variance" then directly gives Eq. (S1.17).

To test whether our model dynamics as described in the Results section of the main text are an artefact of simplified equations regarding the second order moments, we ran a model version that includes corrections for the second order moments (cf. Eq. (S1.16)). The equations for r, g, X, and Y change to:

$$r_a = r + 0.5\, v_\phi \frac{\partial^2 R_X}{\partial \varphi^2} \tag{S1.18}$$

$$g_a = g + 0.5\, v_\omega \frac{\partial^2 g}{\partial \omega^2} \tag{S1.19}$$

$$\frac{dX}{dt} = r_a\, X - g_a\, Y \tag{S1.20}$$

$$\frac{dY}{dt} = (h\, g_a - d)\, Y \tag{S1.21}$$

The model was run with slightly different parameters than the original version (g changed from 1.9 to 2.2, all other parameters as in Table 1). Comparing the model output (Figure S1.2) with the original one (Figure 1, main text) shows that the second order corrections will not lead to substantially new dynamics.

The derivations and tests made in this chapter demonstrate that the moment approximation provides an approximation of an ideal scenario of a larger ensemble of single species (or organisms) each with a fixed and different trait value. In general, it will only slightly differ from this scenario. The major challenge of the derivation thus is the formal effort, but once this is mastered it offers an explicit representation of the functional terms that guide the dynamics in first and second order trait moments (mean value and functional diversity).

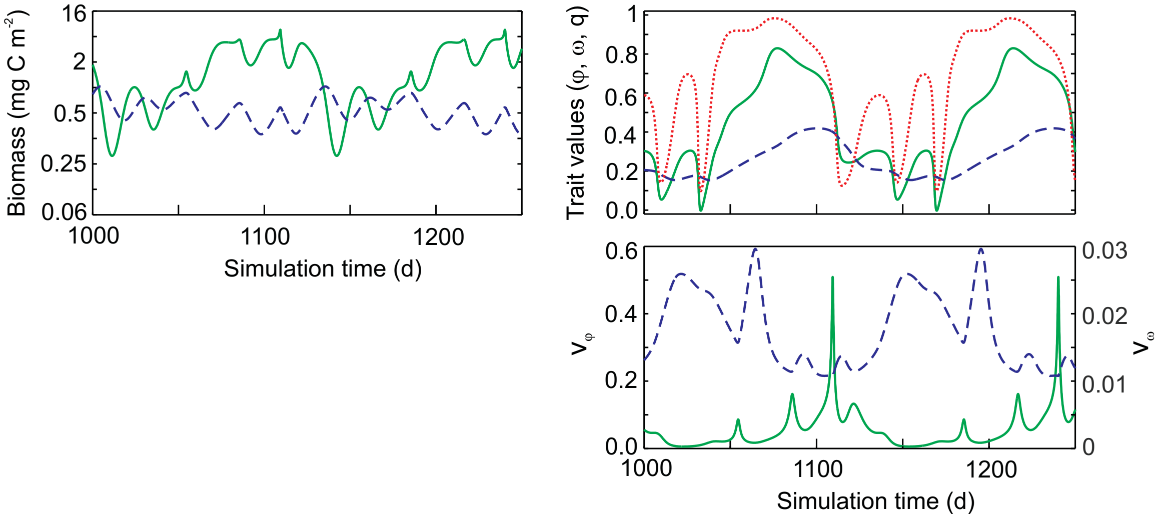

Figure S1.2. Model run with equations corrected for 2nd moments. Simulated prey (green solid) and predator biomass (blue dashed) (left) and the mean trait values, φ (green solid) and ω (blue dashed) and food-suitability q (red dotted), and variance in the φ (green solid) and ω (blue dashed) (right) after a spin-up of 1000 days. Both the prey and the predator have the potential for trait variation. Constants as in Table 1, except g=2.2. Initial conditions for all runs were: $X(0) = 3$, $Y(0) = 1$, $\varphi(0) = 0.54$, $\omega(0) = 0.31$,$v_\phi(0) = v_\omega(0) = 0.06$.

References

1. Beardmore RE, Gudelj I, Lipson DA, Hurst LD (2011) Metabolic trade-offs and the maintenance of the fittest and the flattest. Nature 472: 342–346.

2. Wirtz KW, Eckhardt B (1996) Effective variables in ecosystem models with an application to phytoplankton succession. Ecological Modelling 92: 33-53.

3. Maerz J, Verney R, Wirtz K, Feudel U (2010) Modeling flocculation processes: Intercomparison of a size class-based model and a distribution-based model. Continental Shelf Research doi: 10.1016/j.csr.2010.05.011.
