# Supplementary material for: Predator-Prey Dynamics Driven by Feedback between Functionally Diverse Trophic Levels
Source: PLoS One. 2011 Nov 11;6(11):e27357. doi: 10.1371/journal.pone.0027357 (PMC3214039; doi:10.1371/journal.pone.0027357)
Supplement: Text S2 — Further results of sensitivity analyses concerning the term for the grazing function, growth and grazing parameters, trade-offs, and initial conditions. (PDF) [file pone.0027357.s002.pdf]

Supporting Text S2

Here, we provide further results of sensitivity analyses concerning (1) the term for the grazing function, (2) growth and grazing parameters, (3) trade-offs, and (4) initial conditions.

Grazing function

In the standard version of our model we used a grazing function including a term f_d which reduces grazing at very low prey densities, thus resulting in a sigmoid grazing function [1,2]. Here, we tested the impact of the shape of the grazing function. First, we tested the model with $X_0 = 0$ which results in the 'classical' type-II functional response, i.e. a non-sigmoid function. With the standard parametrization the same dynamics were observed as with $X_0 = 0.02$. Second, we tested the model with a type-III functional response by changing Eq. (8) to

$$g = g_m \frac{(qX)^2}{(qX)^2 + M^2} \tag{S2.1}$$

With the type-III functional response grazing is already strongly reduced at rather low prey biomass which changed the model dynamics remarkably. With the standard parametrization, the predator went extinct. This could be prevented by e.g., changing the predator trade-off function by decreasing b or c. However, we did only observe steady states but no cyclic dynamics.

Growth and grazing parameters

Low maximum prey growth rates ($r_{m0} < 0.2$), low maximum carrying capacities ($K_m < 4$), high minimum half-saturation constants ($M_0 > 1.1$), and low maximum grazing rates ($g_m < 1.1$) led to very low predator biomasses or the extinction of the predator (Figure S2.1 A, B, D; Figure S2.2 A). Extinction of the prey was not observed due to the density dependence of grazing. Very low or zero predator biomass led to an abrupt decline in the edibility of the prey , connected with a higher maximum carrying capacity of the prey (Eq. 15). More generally, relatively high $K_m : M_0$ ratios implying a high maximum carrying capacity or a low minimum half-saturation constant, or low $K_m : M_0$ ratios changed the dynamical behavior to regular predator-prey cycles (Figure S2.1 B, C; Figure S2.2 B, C). The mean trait values of the predator and prey changed little within the parameter range of $r_{m0} > 0.6$, $7 < K_m < 15$, $0.4 < M_0 < 1.1$ and $g_m > 1.4$, although the prey and predator biomasses and their variability showed some differences compared to the standard run. Increasing $r_{m0} > 0.6$ increased the predator, but not the prey biomass (Figure S2.1 A), as the higher prey growth supported higher grazing rates. Increasing the maximum carrying capacity K_m within the range $7 \lesssim K_m \lesssim 15$ resulted in relatively high amplitude cycling of prey and predator (cf. Figure S2.1 B), a behavior in line with the classical effect of enrichment. When raising M_0 from 0.4 to 1.1 prey and predator biomass slightly increased, while their variability decreased (Figure S2.1 C). Increasing g_m above 1.4 resulted in lower average prey and predator biomasses (Figure S2.1 D) and values of $1.4 < g_m < 1.9$ resulted in dynamics similar to that of the standard run, with a weaker decoupling of predator-prey dynamics.

The model is not sensitive to variations in the critical prey density for grazing until unrealistically high values of $X_0 \geq 10$ where the predator went extinct (Figure S2.1 E).

The potential for trait variation at both trophic levels resulted in complex temporal dynamics of the trophic level biomasses for a broad parameter space. This dynamical behavior substantially deviated from predator-prey systems without trait variation. Nevertheless, some overall responses of predator and prey biomasses to changes in the growth and grazing parameters resembled well known properties of classical predator-prey models. For example, enrichment (increasing K_m) or a lower food demand of the predator (decreasing M_0) resulted in a higher temporal variability of predator and prey biomass, and a higher prey growth rate (determined by r_{m0}) increased the predator biomass (cf. [3]).

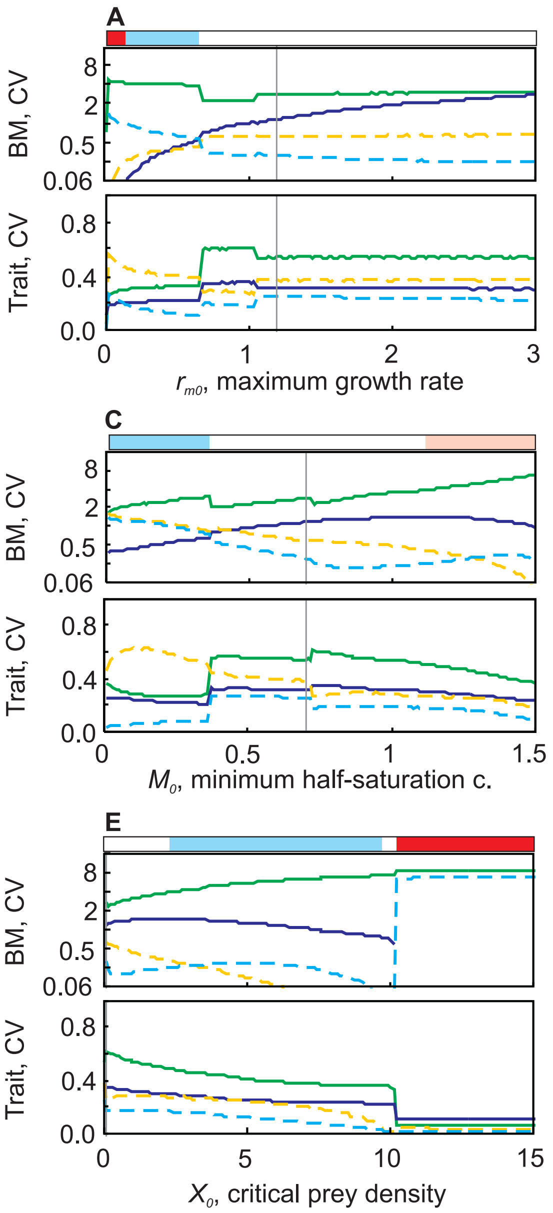

Figure S2.1. Sensitivity of the model behavior. Alterations in: **(A)** maximum growth rate r_{m0}, **(B)** maximum carrying capacity K_m, **(C)** minimum half-saturation constant M_0, **(D)** maximum grazing rate g_m, **(E)** critical prey density X_0. Each panel comprises 2 graphs: The upper graph shows the time averaged prey (green solid line) and predator biomasses (blue solid line) ($\mathrm{g\,C\,m^{-2}}$, log_2 scaled) and the respective CVs (dashed lines, yellow – CV_X, cyano – CV_Y). The lower graph shows the time averaged trait values edibility (green solid line) and food-selectivity (blue solid line) and the respective CVs (dashed lines, yellow – CV_φ, cyano – CV_ω). The vertical lines mark the standard parameter values as given in Table 1. The horizontal bars indicate the dynamics in the predator and prey biomass if they differ from those observed with the standard parametrization (cf. Table 1, Figure 1 D). Red bars indicate the extinction of the predator and a constant biomass of the prey at maximum carrying capacity (for an example see Figure S2.2 A). Light blue bars indicate regular predator-prey cycles, i.e., both predator and prey biomasses oscillate with the same frequency and a constant amplitude, with quarter-period phase-lags (for an example see Figure S2.2 B). Pink bars indicate regular predator-prey cycles with approximately half-period phase-lags (for an example see Figure S2.2 C). See text for details.

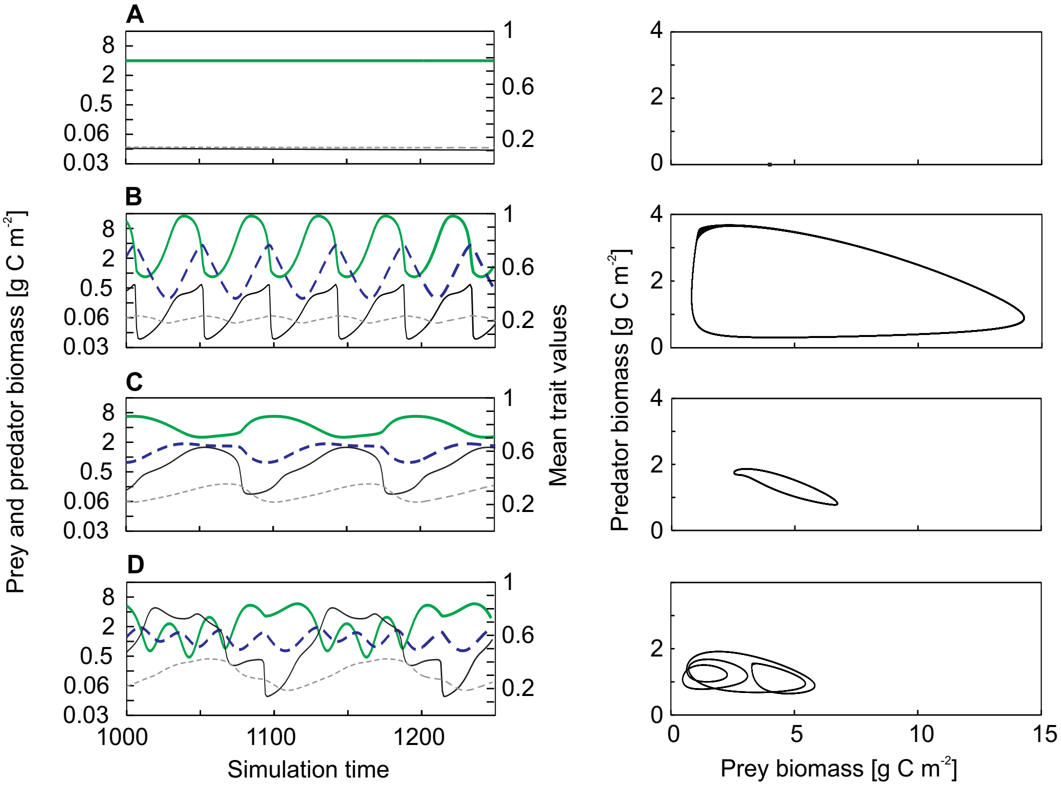

Figure S2.2. Illustration of different types of dynamic patterns obtained at different parametrization. Simulated prey (green solid) and predator biomass (blue dashed) and the respective trait values (black solid for φ and grey dashed for ω; left), and phase portraits of predator and prey biomass (right) after a spin-up of 1000 days. Model run with **(A)** $K_m = 4$, **(B)** $K_m = 18$, **(C)** $M_0 = 1.2$, and **(D)** the standard parametrization ($K_m = 9$, $M_0 = 0.7$). Other constants as in Table 1 .

Trade-offs

We tested the sensitivity of the dynamic-trait model to omitting the trade-off between the carrying capacity K and the prey edibility φ (Eq. 15). A linear relationship between the maximum growth rate r_m and edibility φ led to strongly increasing values of φ as the realized growth rate was not reduced by a decreasing carrying capacity. Coexistence of predator and prey at realistic values of $0 < \varphi < 1$ was possible when changing the shape of the trade-off between r_m and φ (Eq. 14) from linear to concave, and applying a higher value of M_0 to compensate for the overall higher growth rate resulting from the altered trade-off. However, the higher growth and grazing rates resulted in typical predator-prey cycles (similarly to a high $K_m : M_0$ ratio) and low variances of both traits (e.g., low functional diversity). Hence, the trade-off between K and φ was necessary to maintain high functional diversity and substantial oscillations in the trait values.

In addition to the individual effects of a, b and c determining the shape of the trade-offs (Figure 6, cf. main text section 'Sensitivity analysis') we tested which combinations of shapes of the trade-off curves yielded persistent oscillations in both mean trait values, since the different trade-offs are interrelated. In order to identify regions where both traits oscillate simultaneously we used the product $CV_{phi} * CV_{omega}$ as a measure. For a broad range of parameter combinations, the oscillations were sustained. Largely

independent of the values of a and c, values of $b > 8$ (i.e., strong nonlinearity between q and ω) were required for ongoing variations in the mean traits.

Initial conditions

Using the standard parametrization the dynamic-trait model was not sensitive to initial conditions. When running the model with some extreme parameter values (e.g., $K_m > 16$ or $K_m < 4$, or $g_m > 2$) initial conditions gained importance: the shape of the limit cycle of the predator and prey biomasses was either a torus attractor (i.e., complex dynamics similar to that of the standard run, cf. Figure 1 D, Figure S2.2 D) or typical predator-prey cycles occurred (cf. Figure 5 A) or the species ceased to coexist and the predator but not the prey went extinct.

References

1. Baretta-Bekker JG, Baretta JW, Rasmussen EK (1995) The microbial food-web in the european-regional-seas-ecosystem-model. Netherlands Journal of Sea Research 33: 363–379.

2. Tirok K, Gaedke U (2010) Internally driven alternation of functional traits in a multispecies predator-prey system. Ecology 91: 1748-1762.

3. Rosenzweig ML, MacArthur RH (1963) Graphical representation and stability conditions of predator-prey interactions. American Naturalist 97: 209–223.
